# Supplementary figures and images for: 3D fluorescence staining and confocal imaging of low amount of intestinal organoids (enteroids): Protocol accessible to all
Source: PLoS One. 2025 Jan 15;20(1):e0315922. doi: 10.1371/journal.pone.0315922 (PMC11734922; doi:10.1371/journal.pone.0315922)

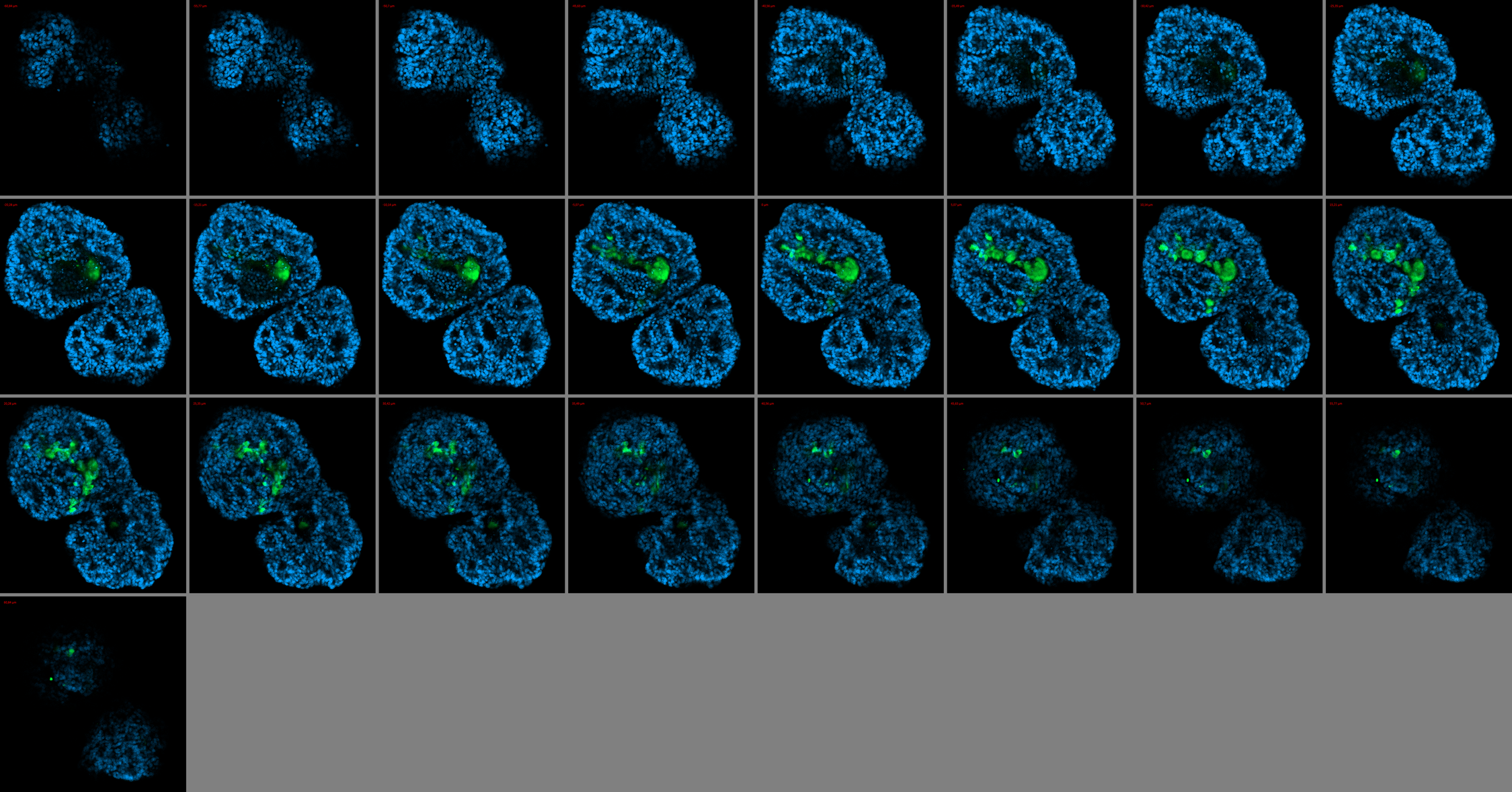

Supplement: S1 Fig — (TIF) [file pone.0315922.s001.tif]

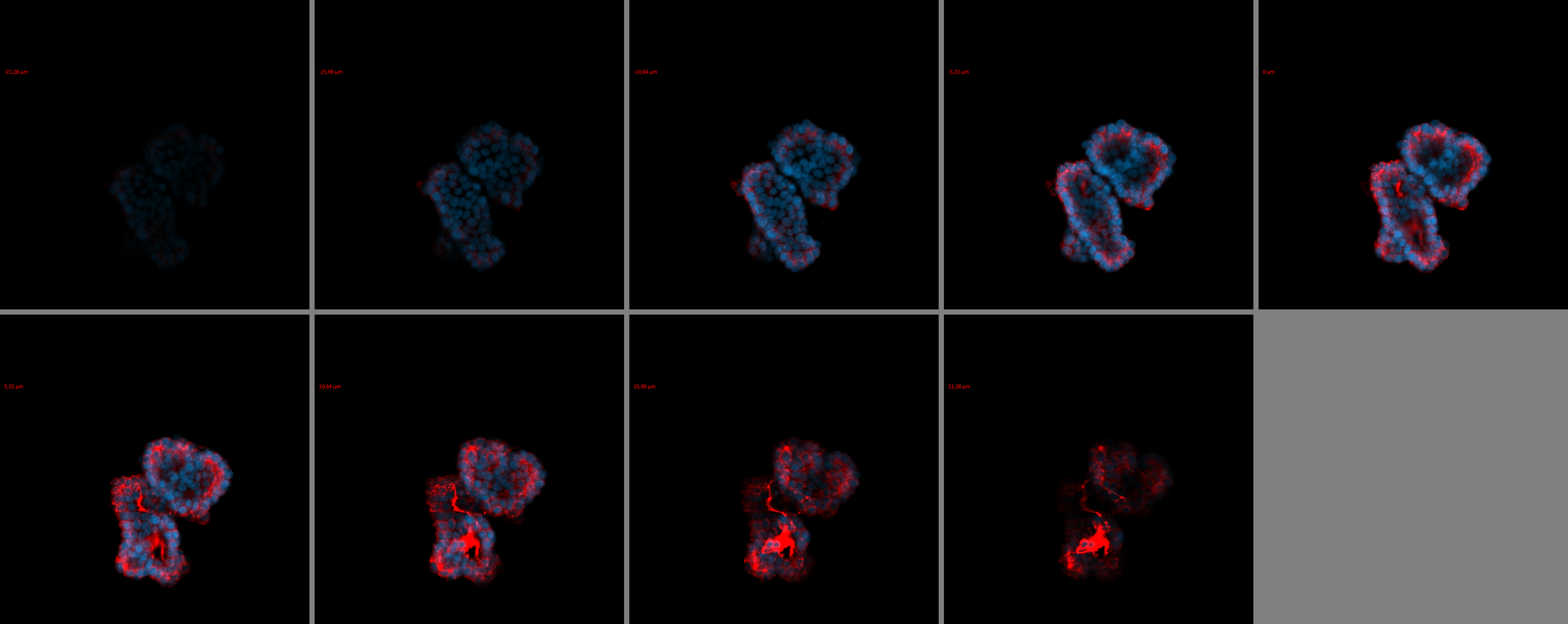

Supplement: S2 Fig — (TIF) [file pone.0315922.s002.tif]

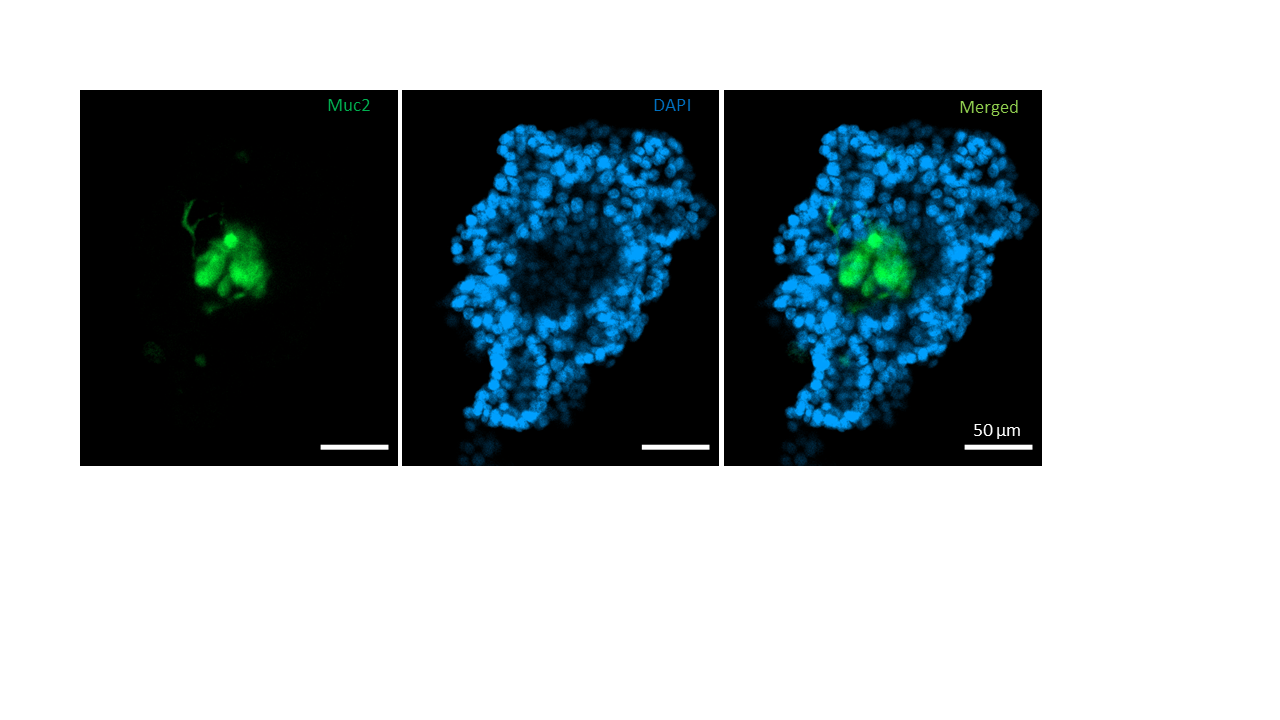

Supplement: S3 Fig — (TIF) [file pone.0315922.s003.tif]
