# Supplementary material for: 3D fluorescence staining and confocal imaging of low amount of intestinal organoids (enteroids): Protocol accessible to all
Source: PLoS One. 2025 Jan 15;20(1):e0315922. doi: 10.1371/journal.pone.0315922 (PMC11734922; doi:10.1371/journal.pone.0315922)
Supplement: S1 File — (PDF) [file pone.0315922.s004.pdf]

3D fluorescence staining and imaging of low amount of organoids

RESERVED DOI:
10.17504/protocols.io.kqdg323m7v25/v1

Ami G. Toulehohoun[1], Carolin Bouzin[2], Aurelie Daumerie[2], Luca Macionni[3], Peter Stärkel[1,4]

[1]Laboratory of Hepato-Gastroenterology, Institut de Recherche Expérimentale et Clinique, Université catholique de Louvain, Brussels, Belgium;
[2]IREC Imaging Platform Belgium (2IP, RRID:SCR_023378), Institut de Recherche Expérimentale et Clinique, Université catholique de Louvain, Brussels, Belgium;
[3]Laboratory of Liver Diseases, National institute of Alcohol Abuse and Alcoholism (NIAAA), The National Institutes of Health (NIH), 5625 Fishers Lanee 20852 Rockvile MD, United States;
[4]Department of Hepato-Gastroenterology, Cliniques Universitaires Saint-Luc, Brussels, Belgium

Ami Gloria TOULEHOHOUN
GAEN/IREC/UCLouvain

Protocol Info: Ami G. Toulehohoun, Carolin Bouzin, Aurelie Daumerie, Luca Macionni, Peter Stärkel . 3D fluorescence staining and imaging of low amount of organoids. **protocols.io** **https://protocols.io/view/3d-fluorescence-staining-and-imaging-of-low-amount-dhf433qw**

Created: July 12, 2024

Last Modified: July 16, 2024

Protocol Integer ID: 103644

Keywords: Organoids, Enteroids, Spheroids, 3D imaging, Confocal microscopy, HIER, Antigen retrieval on organoids

Abstract

The emerging field of 3D organ modeling encounters several imaging issues in particular related to antigen retrieval and sample lost during staining processes. Due to their compact shape, several antibodies fail to penetrate intact organoids or spheroids. Histology of organoids can be approached by paraffin inclusion and sectioning at 5 µm as performed for biopsies. However, to fully understand organoid behavior, including cellular organization, extracellular matrix structure, and their response to treatments, 3D imaging is essential. Here we propose an easy workflow allowing (1) immunostaining with a HIER step, (2) preservation of the intact shape of the organoids, (3) sample immobilization in a focal plane reachable for high resolution/short working distance lenses, and (4) minimizing the risk of loss of precious material.

Guidelines

Introduction:

Organoids are 3D in vitro models, mimicking the native patient organ (healthy or pathologic) in terms of structural and functional aspects. They offer unparalleled potential for disease modelling and large-scale therapeutic screening. Compared to animal models, organoids derived from patients' cells translate into greater similarity in physiological and disease processes and offer a more ethically responsible approach.

Following proper characterization and in vitro expansion, these "mini-tissues" can be established as biobanks, serving as valuable research resources for biomarker identification and novel therapeutic target development. Furthermore, organoid models hold particular promise for studying rare pathologies, where limited access to biological material delays research progress. Their versatility extends to diverse areas, including tumor biology, inflammatory diseases, regenerative medicine, and more. Importantly, unlike formalin-fixed paraffin-embedded tissues, these "living biobanks" provide a source of fresh material suitable for a wide range of analyses, including histological, protein expression, genomic, and metabolomic studies.

Histology of organoids can be approached by paraffin inclusion and sectioning at 5 µm as performed for biopsies (Mahe et al., 2013). However, to fully understand organoid behavior, including cellular organization, extracellular matrix structure, and their response to treatments, 3D imaging is essential. Different imaging technologies allow for 3D imaging of these small (50-300 µm diameter) structures, like light sheet microscopy and confocal microscopy. Light sheet microscopy offers high imaging speed and good spatial resolution (Wan et al., 2019, de Medeiros et al., 2022, Dekkers et al., 2019) while confocal microscopy is the most widely accessible device. Although sample positioning is challenging on both devices, organoid inclusion in agarose sticks for light sheet microscopy leads to a massive sample loss. For this reason, we focused on confocal microscopy.

The emerging field of 3D organ modeling requires adequate sample preparation in order to generate faithful representations of the real 3D structure.

First, fixation with formaldehyde is known to create crosslinking by forming covalent bonds between proteins. This network can also be an obstacle to antibodies penetration. On 2D sections, a heat-induced epitope retrieval step (HIER, at acidic or basic pH) is commonly performed to brake this network and make antigens accessible to antibodies (Dunkenberger et al, 2022). Of note, fixation of organoids with methanol does not require antigen retrieval, yet our prior staining showed that this method is inappropriate for detecting some epitopes and the image quality is lower when compared to fixation performed with formaldehyde. Second, due to their compact shape and thickness, several antibodies fail to penetrate intact organoids. This can be worked around using detergents in the staining protocol. Third, light scattering and/or absorption by biological tissues is an obstacle to in depth imaging. However, optical clearing compounds are now available to render tissues transparent and straighten the light path, improving the thickness of samples that can be imaged in their entirety. Finally, when dispersed in a Matrigel dome, organoids are sometimes out of reach when using lenses with a magnification higher than x10. On the opposite, mounting organoids on a slide with a coverslip at the end of the staining leads to the flattening of the organoids and prevents from imaging them in their native shape.

Here we propose an easy workflow allowing (1) immunostaining with a HIER step, (2) preservation of the intact shape of the organoids, (3) sample immobilization in a focal plane reachable for high resolution/short working distance lenses, and (4) minimizing the risk of loss of precious material (figure 1).

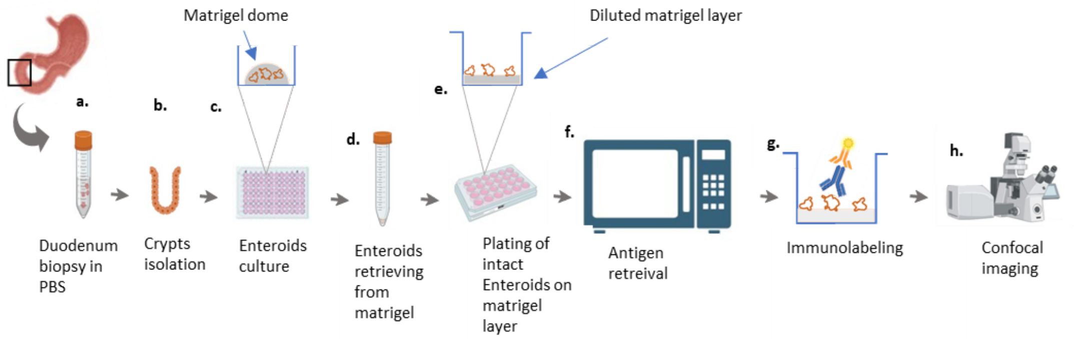

Fig 1. Workflow of organoid formation, staining and imaging. Crypts are isolated (b) from duodenum-derived biopsies (a) and cultured in a Matrigel dome (c). Whole Organoids are retrieved from Matrigel after growth (d) and transferred to Matrigel-coated 24 well plates (e). After attachment, organoids are fixed and submitted to microwave antigen retrieval (f) followed by immunolabeling (g). After clearing, images are acquired by a confocal microscope (g).

Results:

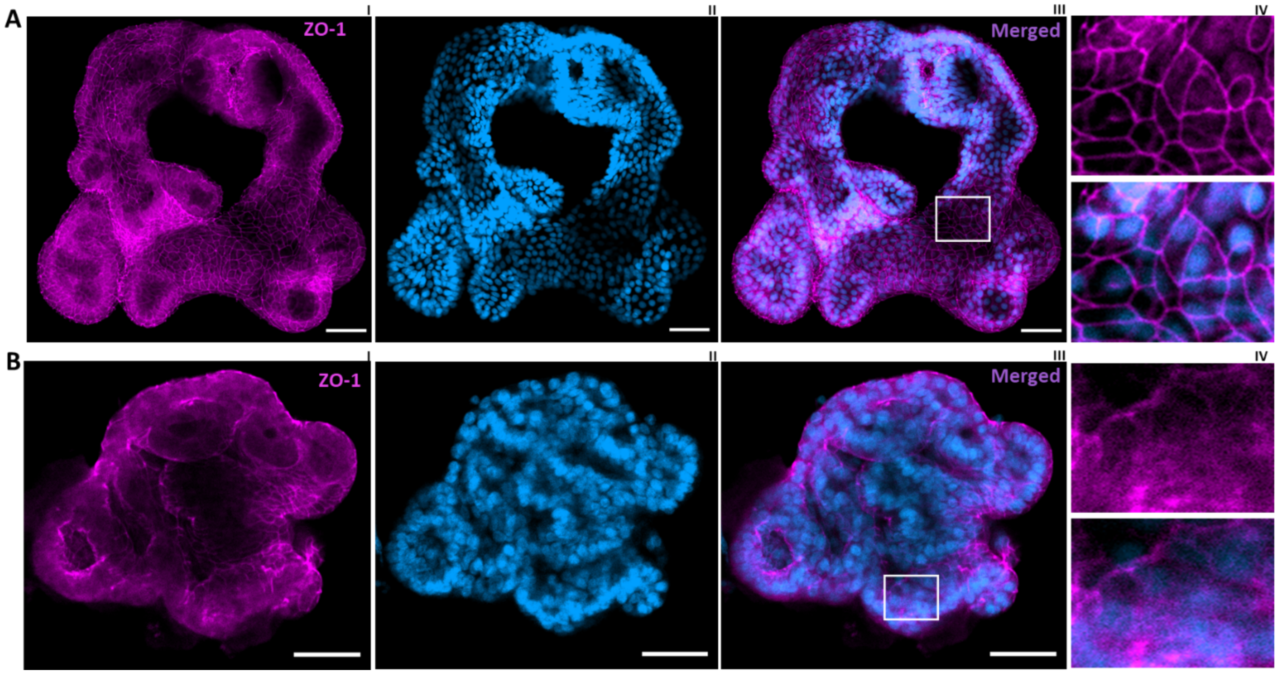

Fig 2. Confocal images of intestinal organoids (enteroids). Staining of tight junctions using Zona occludens 1 (ZO-1) in purple shows specific binding of the antibody to its epitope without antigen retrieval (A) and but not with antigen retrieval (B). DAPI in blue stains the nucleus. AIV & BIV, optical section of indicated volume in AIII and BIII. Scale bar 50 µm

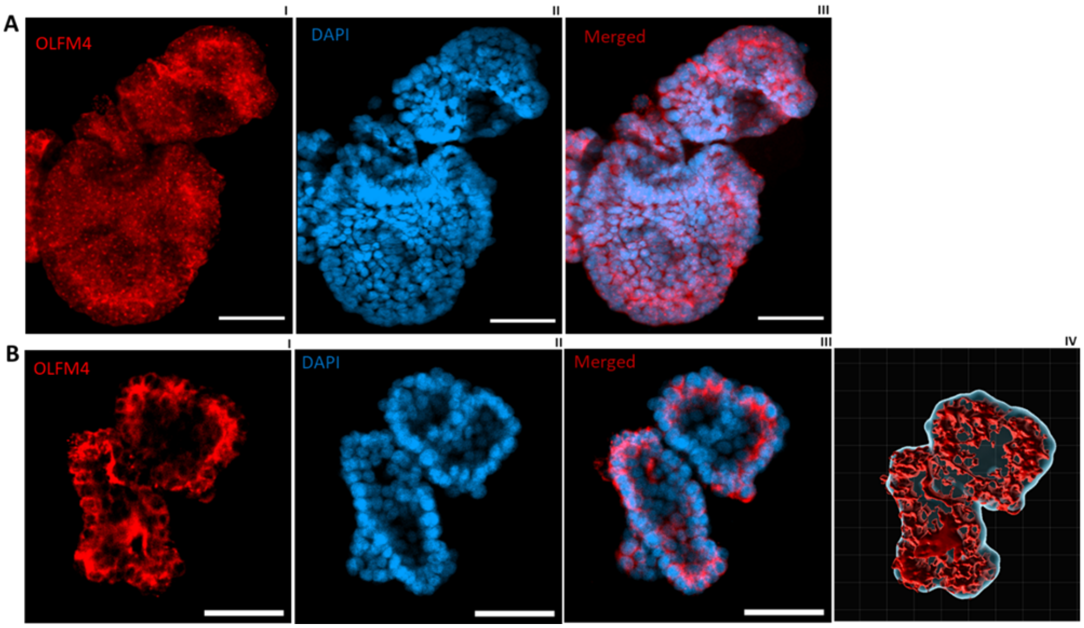
A
OLFM4
I
DAPI
II
Merged
III
B
OLFM4
I
DAPI
II
Merged
III
IV

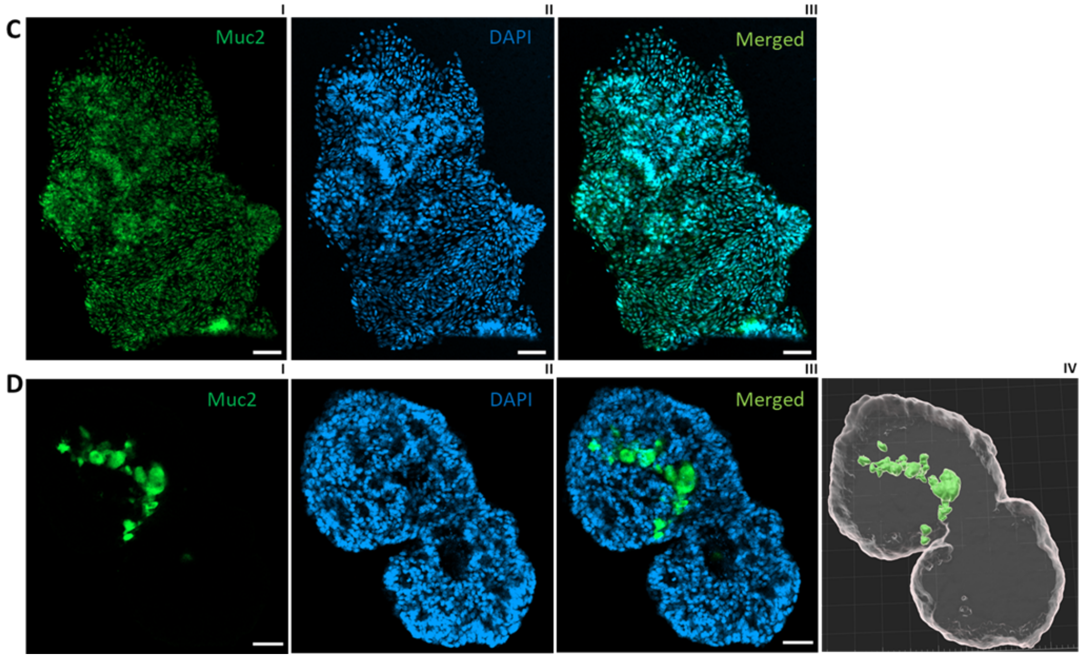
C
Muc2
I
DAPI
II
Merged
III
D
Muc2
I
DAPI
II
Merged
III
IV

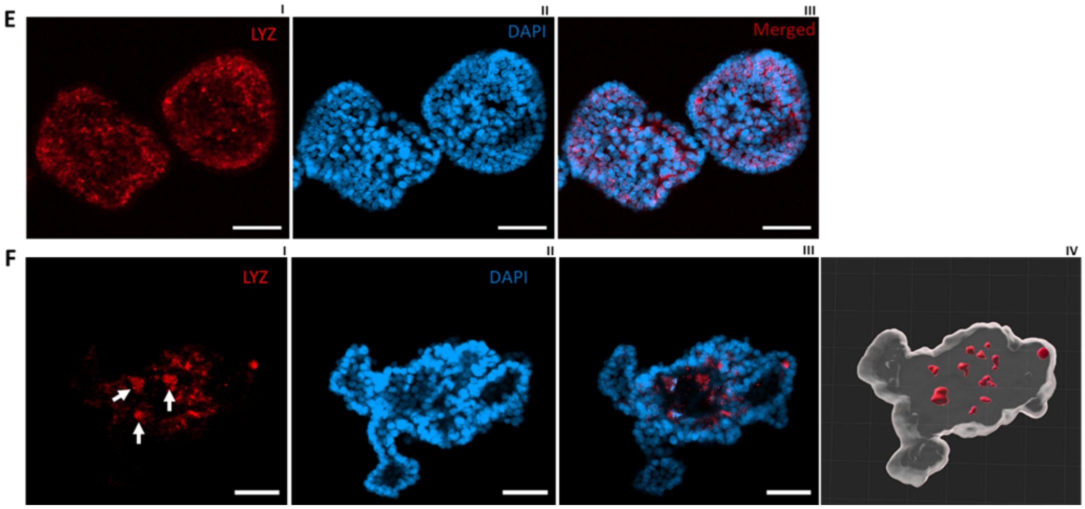

Fig 3. Confocal imaging of Organoids. A. OLFM4 (Olfactomedin 4) in red (A, B), Muc2 (Mucin 2) in green (C, D), Lyz (Lyzozyme) in red (E, F). Unspecific binding of OLFM4, MUC2, and LYZ respectively in (A), (C) and (E) without any prior antigen unmasking. B, D and F, specific binding of OLFM4, Muc2 and Lyz to stem cell and undifferentiated cells, Goblet cells and Paneth cells respectively after antigen retrieval. BIV., DIV and FIV, 3D representations of figures BIII, DIII and FIII respectively. Scale bar 50 µm.

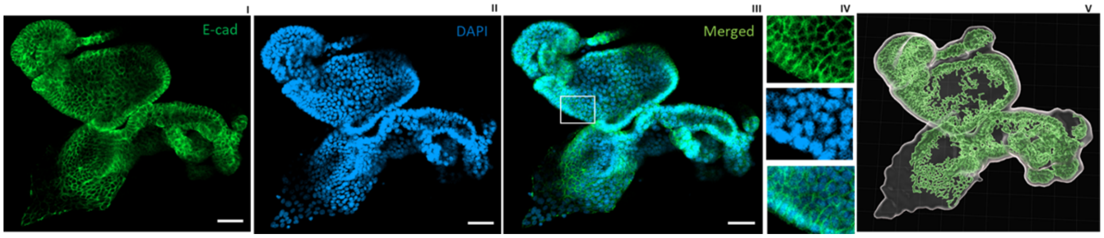

Fig 4. Confocal imaging of Organoids submitted to antigen retrieval. E-cad (membrane protein) in green, marker of epithelial cells. IV., optical sections of indicated volume in III. 3D representation IV. Scale bar 50 µm.

Results and Discussion:

In order to assess the cell components of intestinal organoids, we performed immunostaining to detect MUC2, LYZ, OLFM4, and ZO-1 proteins, respectively expressed by goblet, paneth, stem and stem-like cells and tight junctions. Entire organoids were removed from Matrigel domes and were plated on a diluted Matrigel layer to immobilize them on a single plate for further imaging. Then, they were submitted to fixation and immunostaining without any prior antigen retrieval. They were finally imaged in a clearing solution with a confocal microscope.

Using this protocol, organoids were attached to the plate without forcing or destroying their morphology. Furthermore, after their attachment and fixation, they were tightly stuck to the plate and did not detach during the HIER nor during the washing steps. Consequently, the number of organoids before and after the staining was approximately the same, thus avoiding excessive loss of material.

The cellular localization of a protein of interest is an important element to assess the specificity of an immunostaining. The results represented in figure 2 showed satisfactory tight junction protein ZO-1 detection. However, OLFM4, MUC2 and LYZ staining showed unspecific binding (mainly in the nucleus) of their respective antibodies.

We hypothesized that the lack of specific staining was due to inaccessibility of the antigens. As an antigen retrieval process, we first tried the FLASH technique proposed in a recent publication (Messal et al., 2021). Although this approach might be successful for labeling of some epitopes, it did not allow to detect MUC2, LYZ and OLFM4 proteins in intestinal organoids (data not shown). However, as illustrated in figure 3, we obtained specific staining of Muc2, OLFM4, LYZ protein

in the organoids using our protocol of convenient sample preparation while avoiding sample loss and imaging at the same focal length range.

These results support the added value of our protocol and indicates the need of appropriate antigen retrieval for detecting some epitopes by whole mount immunostaining (al., 2018; Patiño-García et al., 2019; Shi et al., 2011).

Conclusion:

The technique suggested in this protocol allows organoids to attach to the plate while keeping their shape and structure. After fixation, the attached organoids are not removed throughout the whole staining process including Antigen retrieval and washing steps. Moreover, imaging entire organoids by acquiring the different Z-stack positions prevents losing information about each single organoid.

The advantage/ease of this technique is the use of a single plate with different experimental conditions. Moreover, no centrifugation process is required and the washing steps are straight forward.

Limitations:

Compared to previous techniques of sample processing, fixation of the organoids on a plate does not allow Light-sheet microscopy since the organoids are strongly attached to the plate and cannot be removed after the staining. Moreover, Imaging resolution is limited to 10X microscope lenses and 96 well-plates are not suitable for this technique.

Materials

Reagents:

- Dissociation Buffer

Prepare the dissociation buffer by mixing [M] 43.4 millimolar (mM) Sucrose solution (7.43 g in 500 mL distilled water, MW 342.3) and [M] 54.9 millimolar (mM) sorbitol solution (5 g in 500 mL milli Q).

- EDTA [M] 2 millimolar (mM) pH 8

Prepare [M] 0.5 Molarity (M) stock solution by adding 93.05 g of ETDA (MW 372.2) to 500 mL of distilled water. Adjust pH to 8. Dilute 250X the stock solution for ready-to-use solution.

- Citrate buffer

For Antigen retrieval, prepare a 60x concentrated stock solution of citrate buffer with 9.15 g of citric acid (MW: 210.1), 70.35 g tri-sodium dihydrate (MW: 294.1) and 2.4 g Trisma Base (MW: 121.14) in 1 L . Adjust pH to 5.7. storage at 4 °C .

For ready-to-use solution, add 25 mL of citrate buffer stock solution in 1475 mL milli Q water. Add 675 µL of 20X triton X-100 (4 mL of Triton 100 in 16 mL Milli Q water) for each 250 mL 1X citrate buffer.

- PBS-Tween20

Prepare 0.1 % (vol/vol) Tween 20 in PBS by adding 50 µL of Tween-20 in 50 mL PBS.

- PBS-BSA 1%

Add 0.5 g of BSA in 50 mL PBS.

- Blocking and washing buffer

Prepare Organoid Washing Buffer with PBS containing 0.2% BSA and 0.1% Triton X-100 by adding 100 mg BSA and 50 µL Triton X-100 in 50 mL PBS.

Antibodies:

Antibodies	Abbreviation	Dilution	Species	Supplier	Catalogue n°	RRID
Mucin2	MUC2	1:200	Mouse	Santa Cruz	Sc-515032	AB_2815005
Lyzozyme	Lyz	1:400	Rabbit	Abcam	Ab223503	
Olfactomedin 4	OLFM4	1:200	Rabbit	Cell Signaling	14369S	AB_2798465
E-cadherin	E-cad	1:100	Mouse	Dako	M3612	
Zonula Occludens-1	ZO-1	1:100	Rabbit	Invitrogen	617300	
Alexa FluorTM 488 donkey anti-mouse	Anti-Ms	1:1500	Donkey	Invitrogen	A21202	
Alexa FluorTM 488 donkey anti-rabbit	Anti-Rb	1:1500	Donkey	Invitrogen	A21207	

Mucin 2/MUC2 Antibody (F-2) **Santa Cruz Biotechnology Catalog #**sc-515032

Anti-Lysozyme antibody **Abcam Catalog #**ab223503

OLFM4 (D1E4M) XP® Rabbit mAb **Cell Signaling Technology Catalog #**14369S

ZO-1 Polyclonal Antibody **Invitrogen - Thermo Fisher Catalog #**61-7300

anti-Mouse **Invitrogen - Thermo Fisher Catalog #**A21202

Equipments:

- Consumables

1. 96 well plates, Greiner (G) Bio-one, CELLSTAR® cat N° 655180
2. 24 well plates, Greiner (G) Bio-one, CELLSTAR® cat N° 62210
3. 15 ml conic tubes, Greiner (G) Bio-one, CELLSTAR® 88271N

- Bench apparatus

1. Microwave oven
2. Incubator
3. Centrifuge

- Microscopes

The images were acquired by a Zeiss LSM800 inverted confocal microscope equipped with 4 lasers (405, 488, 561 and 640 nm), Variable Secondary Dichroics, GaAsp detectors and x10/NA 0.3 Plan-Apochromat lens.

- Software

Zen 3.5 (blue edition) and Imaris (Bitplane) were used for 3D reconstitution of the images acquired by a confocal microscope.

Antibiotic-Antimycotic (100X) **Thermo Fisher Scientific Catalog #**15240062

Corning® Matrigel® Growth Factor Reduced (GFR) Basement Membrane Matrix, phenol red-free **VWR International Catalog #**734-1101

IntestiCult™ Organoid Growth Medium (Human) **STEMCELL Technologies Inc. Catalog #**06010

Cell Recovery Solution, Corning® **VWR International Catalog #**734-0107

TrypLE™ Express Enzyme (1X), phenol red **Thermo Fisher Catalog #**12605010

Corning® Cell Recovery Solution **Corning Catalog #**CLS354253

Crypt isolation ○ Timing 3h

1h 21m 10s

1

Note

All the following steps are performed at 4 °C On ice .

Collect duodenum biopsies in sterile PBS (without Ca2+ or Mg2+).

2 Wash 3x2 min under mild rotation in PBS + antibiotics and antimycotic 100X (Invitrogen, 15240062, 1/200). (1/3)

2.1 Wash 00:02:00 under mild rotation in PBS + antibiotics and antimycotic 100X (Invitrogen, 15240062, 1/200). (1/3) 2m

2.2 Wash 00:02:00 under mild rotation in PBS + antibiotics and antimycotic 100X (Invitrogen, 15240062, 1/200). (2/3) 2m

2.3 Wash 00:02:00 under mild rotation in PBS + antibiotics and antimycotic 100X (Invitrogen, 15240062, 1/200). (3/3) 2m

3 Incubate for 01:00:00 in [M] 2 millimolar (mM) EDTA in a 15 ml conic tube under slow rotation. 1h

Note

This step aims to loosen (detach?) the crypts from the intestinal mucosa.

4 Allow biopsies to settle down (00:00:10) and discard the supernatant. 10s

5 Add 1 mL chelation buffer (43.4 mM sucrose + 54.9 mM D-sorbitol in DPBS) and transfer into 2 ml cryotube.

Note

This step allows trapping of ions involved in molecular enzymatic degradation of the crypts

6 Shake vigorously by hand. Allow biopsies to settle down (… min) and collect the supernatant containing crypts in a 15 ml conic tube. The remaining biopsies will sediment in the cryotube.

7 Repeat the previous step twice or three times to ensure total recovery of crypts.

8 Add 5 mL of DMEM medium supplemented with 1% Penicillin/Streptomycin and 20% FBS.

9 Centrifuge at 400 x g, 4°C, 00:08:00 . 8m

10 Discard the supernatant.

11 Wash once in 2 mL DMEM medium.

12 Filter through 70 µm cell strainer.

13 Centrifuge at 400 x g, 4°C, 00:07:00 . 7m

14 Discard the supernatant.

15 Resuspend the pellet in 20 µL - 30 µL cold Corning® Matrigel® Growth Factor Reduced (GFR) Basement Membrane Matrix, Phenol Red-free (VWR, 734-1101) and plate 25 µL - 30 µL per well in flat bottom 96 well plate (96 well plates, Greiner (G) Bio-one, CELLSTAR® cat N° 655180)

16 Incubate at 37 °C , 5% CO_2 for 00:10:00 to allow the Matrigel to polymerize. 10m

17 Add 37 °C heated IntestiCult OGM Human (STEMCELL technologies, #06010) containing 1% Penicillin/Streptomycin.

Organoid passaging and culture ○ Timing 7-14d

1h 19m

18 Remove medium from the Matrigel dome.

19 Add 200 µL /well of Corning® Cell Recovery Solution (VWR, #734-0107).

20 Incubate On ice for 00:40:00 .
40m

21 Pull the enteroids into a 15 ml conic tube.

22 Centrifuge at 400 x g, 00:07:00 .
7m

23 Discard the supernatant.

24 Wash once with 2 mL cold DMEM medium.

25 Centrifuge 400 x g, 4°C, 00:07:00 .
7m

26 Discard the supernatant.

27 Add 2 mL Gibco™ TrypLE™ Express Enzyme (1X), phenol red (Thermofisher, #12605010) to the pellet.

28 Incubate for 00:25:00 at 37 °C .
25m

29 Neutralize with 3 mL DMEM medium containing FBS (1/20).

30 Pipette up and down 10 times to dissociate remaining organoids clusters.

31 Centrifuge as previously and discard the supernatant.

32 Wash once with DMEM.

33 Resuspend the pellet in cold Matrigel and plate approximately 15000 single cells in 20 µL - 30 µL per well in a flat bottom 96 well plate (Greiner (G) Bio-one, CELLSTAR ® cat N° 655180).

Note

#TIPS: All organoid are not dissociated into single cells. Some cluster or 3-5 cells could remain.

34 Add heated IntestiCult OGM Human (STEMCELL technologies, #06010) containing 1% Penicillin/Streptomycin.

35 Change medium every 2 days until day 6.

36 Change medium every day from day 7.

Organoid preparation for imaging

1h

37 **Plate coating ○ Timing** 00:30:00 30m

Dilute Matrigel 50x with cold PBS.

38 Plate 200 µL /well of diluted Matrigel in a 24 well plate (Greiner (G) Bio-one, CELLSTAR ® cat N° 62210).

Note

CRITICAL STEP:

1. A thin homogeneous coating of Matrigel is critical to allow organoids attachment in a similar plane.
2. If high resolution is needed, select plates with coverslip bottom

39 Incubate at least 00:20:00 - 00:30:00 at 37 °C to allow the Matrigel to polymerize. 30m

Enteroids retrieving ○ Timing 3h

2h 47m

40 Remove culture medium from the organoids.

41 Add 200 µL /well of Corning® Cell Recovery Solution (CRC, 354253).

42 Place the plate On ice .

43 Incubate for 00:40:00 On ice . 40m

44 Transfer the detached organoids in a 15 ml conic tube.

45 Wash each well 2X with Cell Recovery Solution and add this to the 15 ml tube.

46 Centrifuge at 400 x g, 4°C, 00:07:00 . 7m

47 Discard the supernatant.

48 Wash 1x with DMEM medium.

49 Centrifuge as previously and discard the medium.

50 Add 200 µL /well culture medium on each pellet (containing treatment if applicable).

Note

# TIPS: Calculate the adequate volume depending on the total number of wells

51 Remove the 24 well coated plate from the incubator and remove the excess PBS with 200 µl tips.

52 Plate 200 µL /well of cell suspension.

53 Incubate 01:30:00 - 02:00:00 at 37 °C to allow the organoids to attach to the plate. 2h

Fixation ○ Timing 1h

45m

54 Remove the excess of medium.

55 Wash 1x with heated PBS (37 °C).

56 Fix with cold 4% formaldehyde for 00:45:00 at 4 °C . 45m

57 Wash 1x with cold PBS (4 °C).

Note

# TIPS: Samples can be stored in PBS at 4 °C , before being processed
! CAUTION Manipulate the formaldehyde under the hood.

58 Proceed directly to permeabilization if the antibody does not require an antigen retrieval step.

Antigen retrieval, permeabilization, blocking ○ Timing 1 day

8h 30m 30s

59 Remove PBS.

60 **Prepare 250 ml of citrate buffer:**
Add 25 mL of citrate buffer stock solution 10x concentrated (3.78 g citric acid and 24.12 g sodium citrate dihydrate in 100 mL distilled water, pH adjusted at 5.6, volume adjusted at 1 L , storage at 4 °C) and 675 µL of 20% Triton-X100 solution (diluted in distilled water) in 225 mL distilled water.

61 Fill each well with citrate buffer at Room temperature .

62 Boil the remaining buffer in microwave at 900 Watt and keep it aside.

63 Put the plate in the microwave.

64 Heat at 900W until boiling (approximately 00:00:30). 30s

65 Replace citrate buffer by hot citrate buffer.

66 Repeat three times by filling the well before each boiling.

67 Heat at 90 Watt for 00:15:00 . 15m

68 Boil 3 times at 900 Watt.

69 Let the plate cool down for 00:10:00 - 00:15:00 . 15m

70 Wash 1X with distilled water.

71 Permeabilize and Block with Organoid Washing Buffer (OWB) Overnight (Dekkers et al., 2019). 8h

Note

OWB : 1 ml of Triton X-100 and 2g of BSA to 1l of PBS.

Antibody staining ○ Timing 2 days

9h 25m

72 Dilute primary antibody in OWB solution.

73 Incubate organoids with 300 µL of primary antibody solution Overnight at 4 °C . 8h

74 Wash 2x 5 min with 500 µl OWB.

74.1 Wash 00:05:00 with 500 µL OWB. (1/2) 5m

74.2 Wash 00:05:00 with 500 µL OWB. (2/2) 5m

75 Incubate with 300 µL of secondary antibody in OWB solution Overnight at 4 °C . 15m

76 Wash twice with OWB as described previously.

77 Incubate with 300 µL of DAPI (ref, stock 1µg/ml diluted 1:1000) for at least 01:00:00 . 1h

78 Wash twice with OWB.

79 Wash with distilled water.

80 Remove completely the water.

Clearing and imaging

2h

81 Add 3 drops/well of RapiClear 1.49 (Sunjin Lab #RC147001).

> **Note**
>
> **CRITICAL STEP** Without clearing, only the external cell layers are detectable.

82 Image after 02:00:00 or the next day. 2h

Protocol references

References:

1. Mahe, M. M., Aihara, E., Schumacher, M. A., Zavros, Y., Montrose, M. H., Helmrath, M. A., Sato, T., & Shroyer, N. F. (2013). Establishment of Gastrointestinal Epithelial Organoids. In Current Protocols in Mouse Biology (Vol. 3, Issue 4). https://doi.org/10.1002/9780470942390.mo130179

2. Wan, Y., McDole, K., & Keller, P. J. (2019). Light-sheet microscopy and its potential for understanding developmental processes. Annual Review of Cell and Developmental Biology, 35, 655–681. https://doi.org/10.1146/annurev-cellbio-100818-125311

3. de Medeiros, G., Ortiz, R., Strnad, P., Boni, A., Moos, F., Repina, N., Challet Meylan, L., Maurer, F., & Liberali, P. (2022). Multiscale light-sheet organoid imaging framework. Nature Communications, 13(1), 1–14. https://doi.org/10.1038/s41467-022-32465-z

4. Dekkers, J. F., Alieva, M., Wellens, L. M., Ariese, H. C. R., Jamieson, P. R., Vonk, A. M., Amatngalim, G. D., Hu, H., Oost, K. C., Snippert, H. J. G., Beekman, J. M., Wehrens, E. J., Visvader, J. E., Clevers, H., & Rios, A. C. (2019). High-resolution 3D imaging of fixed and cleared organoids. Nature Protocols, 14(6), 1756–1771. https://doi.org/10.1038/s41596-019-0160-8

5. Dunkenberger L, Del Valle L. Antigen Retrieval and Signal Amplification. Methods Mol Biol. 2022; 2422:65-74. doi: 10.1007/978-1-0716-1948-3_5. PMID: 34859399.

6. Messal, H. A., Almagro, J., Zaw Thin, M., Tedeschi, A., Ciccarelli, A., Blackie, L., Anderson, K. I., Miguel-Aliaga, I., van Rheenen, J., & Behrens, A. (2021). Antigen retrieval and clearing for whole-organ immunofluorescence by FLASH. Nature Protocols, 16(1), 239–262. https://doi.org/10.1038/s41596-020-00414-z

7. Krenacs, L., Krenacs, T., Stelkovics, E., & Raffeld, M. (2010). Heat-induced antigen retrieval for immunohistochemical reactions in routinely processed paraffin sections. Methods in molecular biology (Clifton, N.J.), 588, 103–119. https://doi.org/10.1007/978-1-59745-324-0_14

8. Citi, S., Fromm, M., Furuse, M., González-Mariscal, L., Nusrat, A., Tsukita, S., & Turner, J. R. (2024). A short guide to the tight junction. Journal of Cell Science, 137(9). https://doi.org/10.1242/jcs.261776

9. Patiño-García, D., Rocha-Pérez, N., Moreno, R. D., & Orellana, R. (2019). Antigen retrieval by citrate solution improves western blot signal. MethodsX, 6, 464–468. https://doi.org/10.1016/j.mex.2019.02.030

10. Shi, S. R., Shi, Y., & Taylor, C. R. (2011). Antigen retrieval immunohistochemistry: Review and future prospects in research and diagnosis over two decades. Journal of Histochemistry and Cytochemistry, 59(1), 13–32. https://doi.org/10.1369/jhc.2010.957191
