# Supplementary material for: 3D fluorescence staining and confocal imaging of low amount of intestinal organoids (enteroids): Protocol accessible to all
Source: PLoS One. 2025 Jan 15;20(1):e0315922. doi: 10.1371/journal.pone.0315922 (PMC11734922; doi:10.1371/journal.pone.0315922)
Supplement: Attachment — Submitted filename: Minor revisions.pdf [file pone.0315922.s005.pdf]

Minor revisions:

The results and discussion section is lacking. There is no reference to Fig 4.

Line 17. related to antigen retrieval and sample ~~lost~~ *loss* during staining processes. Due to their compact

Line 85. retrieval (A) ~~and~~ but not with antigen retrieval (B). DAPI in blue stains the nucleus. AIV & BIV, optical

Line 94. green, marker of epithelial cells. IV., optical sections of indicated volume in III. 3D representation IV. *In Fig 4 there is one more picture magnification (V)*

Line 121. These results support the added value of our protocol and *indicate* the need of appropriate

Line 146. Shake vigorously by hand. Allow biopsies to settle down *(?min)* and collect the
