# Supplementary material for: 3D fluorescence staining and confocal imaging of low amount of intestinal organoids (enteroids): Protocol accessible to all
Source: PLoS One. 2025 Jan 15;20(1):e0315922. doi: 10.1371/journal.pone.0315922 (PMC11734922; doi:10.1371/journal.pone.0315922)
Supplement: Attachment — Submitted filename: Response to reviewers.docx [file pone.0315922.s006.docx]

Reviewers' comments:

Reviewer's Responses to Questions

**Comments to the Author**


1. Does the manuscript report a protocol which is of utility to the research community and adds value to the published literature?

Reviewer #1: No

Reviewer #2: Yes

Reviewer #3: Yes

Reviewer #4: Yes

Reviewer #5: No

Reviewer #6: No

Reviewer #7: Yes

2. Has the protocol been described in sufficient detail?


To answer this question, please click the link to protocols.io in the Materials and Methods section of the manuscript (if a link has been provided) or consult the step-by-step protocol in the Supporting Information files.


The step-by-step protocol should contain sufficient detail for another researcher to be able to reproduce all experiments and analyses.

Reviewer #1: No

Reviewer #2: Yes

Reviewer #3: Yes

Reviewer #4: Yes

Reviewer #5: Yes

Reviewer #6: Yes

Reviewer #7: Yes

3. Does the protocol describe a validated method?

The manuscript must demonstrate that the protocol achieves its intended purpose: either by containing appropriate validation data, or referencing at least one original research article in which the protocol was used to generate data.

Reviewer #1: No

Reviewer #2: Yes

Reviewer #3: Yes

Reviewer #4: Yes

Reviewer #5: Yes

Reviewer #6: Yes

Reviewer #7: No

4. If the manuscript contains new data, have the authors made this data fully available?

The [PLOS Data policy](https://eur03.safelinks.protection.outlook.com/?url=http%3A%2F%2Fc05y1x9s.r.us-east-2.awstrack.me%2FL0%2Fhttp%3A%252F%252Fwww.plosone.org%252Fstatic%252Fpolicies.action%2523sharing%2F1%2F010f01929b69d353-0606ddd9-22f7-467d-8997-ba2f3f045560-000000%2FjqKn5mDl09qXmo9m5Y_og0wtgG4%3D181&data=05%7C02%7Cami.toulehohoun%40uclouvain.be%7Cfdc26f27d0864089978a08dcf32cb8a1%7C7ab090d4fa2e4ecfbc7c4127b4d582ec%7C1%7C0%7C638652620611067204%7CUnknown%7CTWFpbGZsb3d8eyJWIjoiMC4wLjAwMDAiLCJQIjoiV2luMzIiLCJBTiI6Ik1haWwiLCJXVCI6Mn0%3D%7C0%7C%7C%7C&sdata=fOCrezjQWpplqDQ2CEiVAE9enYDsO69q%2BcdlDwrAOjg%3D&reserved=0) requires authors to make all data underlying the findings described in their manuscript fully available without restriction, with rare exception (please refer to the Data Availability Statement in the manuscript PDF file). The data should be provided as part of the manuscript or its supporting information, or deposited to a public repository. For example, in addition to summary statistics, the data points behind means, medians and variance measures should be available. If there are restrictions on publicly sharing data—e.g. participant privacy or use of data from a third party—those must be specified.

Reviewer #1: No

Reviewer #2: Yes

Reviewer #3: No

Reviewer #4: Yes

Reviewer #5: No

Reviewer #6: N/A

Reviewer #7: N/A

**5. Is the article presented in an intelligible fashion and written in standard English?**


PLOS ONE does not copyedit accepted manuscripts, so the language in submitted articles must be clear, correct, and unambiguous. Any typographical or grammatical errors should be corrected at revision, so please highlight any specific errors that need correcting in the box below.

Reviewer #1: **No:** must need proof reading services

Reviewer #2: Yes

Reviewer #3: Yes

Reviewer #4: Yes

Reviewer #5: Yes

Reviewer #6: **No:** There are spelling mistakes and English grammar errors. Editing is recommended.

Reviewer #7: Yes

6. Review Comments to the Author

Please use the space provided to explain your answers to the questions above. You may also include additional comments for the author, including concerns about dual publication, research ethics, or publication ethics. (Please upload your review as an attachment if it exceeds 20,000 characters)

**Reviewer #1**: The manuscript was poorly written, and the authors failed to convince that the method they developed was reliable and repeatable based on what was presented here.

The heat-induced method of epitope retrieval was a well-established technique routinely used for histological staining.

We agree with the reviewer that Heat induced epitope retrieval (HIER) is indeed routinely used for 2D FFPE sections. However, this cannot be extrapolated to 3D staining and, to the best of our knowledge, HIER is not well established for 3D staining due the complexity of the samples management. This sentence has been added in the introduction. **L58-L61**

The sandwich method of placing organoids and paraformaldehyde fixation is recommended by Corning and routinely used in organoid labs. Hence, I do not see novelty in their study.

In the Corning "sandwich method", the Matrigel is dispensed into the plate and is allowed to polymerize. Next, the organoid cell suspension is mixed with a dilution of the matrix and dispensed on top of the polymerized layer and is incubated for an additional duration.

In our protocol, pre-formed organoids (not cell suspension) are seeded on a layer of a polymerized Matrigel. They are not embedded in an additional Matrigel layer and hence directly accessible to antibodies with an accessible working distance from the objectives.

([https://www.corning.com/worldwide/en/products/life-sciences/resources/stories/the-cutting-edge/going-tiny-is-the-next-big-thing-tools-and-techniques-for-organoid-cultures.html](https://eur03.safelinks.protection.outlook.com/?url=https%3A%2F%2Fwww.corning.com%2Fworldwide%2Fen%2Fproducts%2Flife-sciences%2Fresources%2Fstories%2Fthe-cutting-edge%2Fgoing-tiny-is-the-next-big-thing-tools-and-techniques-for-organoid-cultures.html&data=05%7C02%7Cami.toulehohoun%40uclouvain.be%7Cfdc26f27d0864089978a08dcf32cb8a1%7C7ab090d4fa2e4ecfbc7c4127b4d582ec%7C1%7C0%7C638652620611093781%7CUnknown%7CTWFpbGZsb3d8eyJWIjoiMC4wLjAwMDAiLCJQIjoiV2luMzIiLCJBTiI6Ik1haWwiLCJXVCI6Mn0%3D%7C0%7C%7C%7C&sdata=1NyWWVNhhT74NbKHZHvE32786jiOU8OZ0FvgxZpatc0%3D&reserved=0))

This point has been highlighted in the discussion: “In this protocol, the pre-formed enteroids are not embedded in the Matrigel and hence are directly accessible to antibodies with an accessible working distance from the objectives.” **L250-L252**

In the abstract, the authors mentioned that 3D imaging is necessary but failed to show 3D imaging techniques such as Light-sheet microscopy.

We totally agree that Light-sheet imaging is a technique of choice for 3D imaging. It is much faster than confocal and has a very good axial resolution. However, when using samples as small as organoids, many of them are lost during the mounting steps in the agarose necessary to position them in the imaging chamber. For this reason, we did not find it suitable for small amount or tiny samples. Moreover, confocal imaging in multiwell plates allows an easy shift from growth follow-up with plate readers.

I think the authors should have at least shown a confocal-built 3D image.

Fig3 B IV, D IV, F IV and Fig4 display a 3D reconstruction and supplementary figures (S1-Fig & S2-Fig) have been added showing the different acquisition layers (z-stacks).

It is well known, and even described in the Matrigel manual, that the proteins in Matrigel can autofluorescence.

Signal to noise ratio was sufficient to unambiguously discriminate the stainings, probably owing to the thin coating layer of Matrigel.

Lastly, when the authors claim that they developed an easy workflow for staining, they should have compared their method with a more difficult workflow and summarized their results in a table/figures.

Our method has been compared to Light-sheet workflow since we started the organoids staining and imaging by Light-sheet microcopy before shifting to a more convenient and easier workflow as described in our protocol.

This has been mentioned in the introduction **L52-L56**: “Light sheet microscopy offers high imaging speed and good spatial resolution (Wan et al., 2019, de Medeiros et al., 2022, Dekkers et al., 2019) while confocal microscopy is the most widely accessible device. Although sample positioning is challenging on both devices, organoid inclusion in agarose sticks for light sheet microscopy leads to a massive sample loss. For this reason, we focused on confocal microscopy.”

Comments:
1. Line 28: “mimicking patient…”, why not animals? Organoids were generated in animals; re-phrase this statement.

Organoids can indeed be derived from animals. Since no good animal model recapitulating changes seen in humans in alcohol research does exist, it seemed evident to us to start with patients’ samples and directly develop patient-derived organoids that could mimick the human condition. Detailed discussion why patients and not animals goes too far beyond the scope of a descriptive protocol.
2. Figure 3: I can't find all the images described in the figure legend

It is our mistake and we apologize for this omission. We now up-loaded separately Figures 3A-B, C-D and E-F.
3. Table 1 should include conc. , not dilutions of antibodies.

It has been adapted. Concentrations are now included in Table 1.
4. Must add more references for some of the statements quoted, e.g. lines 54-55

A reference has been added. **L63**
5. Line 139: what is 1/200?

It has been corrected **L106**

6. Line 143: what is a chelation buffer? I think there may be additional components in this buffer.

This buffer has been described in the reagents section and the name has been changed to dissociation buffer **L295**
7. Line 146: “…min)?

It has been filled in. **L113**

8. Line 200: Which culture medium?

IntestiCult OGM Human. This has been included. **L166**
9. Line 214: Once Matrigel is polymerized, how can the organoids attach?

Matrigel thin layer in this case acts as a coating (like poly-L-lysin or gelatin)
10. Line 270: Compared to which techniques??

We rephrased this sentence. **L288**

11. It looks like a copy-paste lab protocol, not proofread

This protocol has been written according to PLOS One lab protocol guidelines. We apologize that it contained some grammatical errors that have been corrected by a native speaker.

12. Inconsistency in describing the vendors or manufacturer throughout the manuscript. Corning is the manufacturer, not VWR

This has been corrected. **L133**
13. It is interesting to see only one manuscript referenced from Dr. Clever's lab

We added an additional review citation from Dr. Clever Hans in the introduction. **L35**
14. This manuscript requires thorough proofreading for grammar, spell-checks, font sizes, and inconsistency.

The manuscript has been proofread by a native speaker and errors have been corrected

**Reviewer #2**: This protocol described how to stain and image 3D organoids. Some questions are:
1. Please state the confocal microscope in the title, since 3D imaging could be achieved by other ways, for example fluorescent MOST.

The title has been adapted to: "3D fluorescence staining and confocal imaging of low amount of intestinal organoids (enteroids): protocol accessible to all"

2. In looking at the presented images (2-4), it seems that they are not like 3D imaging, because they do not show the whole organoids. Did the authors only image a few layers, not the whole organoids?

Entire organoids have been acquired but for visualization purposes, only the representative central layer is shown.

In case the complete figures have not been adequately up-loaded during our submission, we now up-loaded separately Figures 3A-B, C-D and E-F.

Fig3 B IV, D IV, F IV and Fig4 display a 3D reconstruction and supplementary figures (S1 Fig and S2 Fig) have been added showing the different acquisition layers (z-stacks).

3. How low is low amount?

In our experiments, we used as few as 10 grown organoids. This information has been integrated in the conclusion section. **L285**

4. Please state the types of organoids. Since they are from crypts, they should be intestine organoids. Then could this protocol be used to other organoids, such as brain organoids, since brain organoids contain more contact core.

Yes, these organoids have been formed from crypts. In the literature, such organoids are called enteroids. The title has been adjusted according to this comment. This protocol can be used for other types of organoids. In fact, tissue dissociation requires organ-specific dissociation solutions. But the staining/imaging workflow described in this article should be applicable to any organoid type. Since we do not show here other organoids, we added this as a limitation of this study. **L291-L292**

**Reviewer #3:** The protocol is easy to comprehend and follow, but requires some fine tuning to be more applicable for efficient visualization at a higher magnification.
These are some minor comments:
There are some incomplete sentences in the methods (L141 and L146). Please revise and double-check.

This has been corrected. **L108, L113**
For each image and in the protocol, please specify on which day of culture you sampled the organoids (day 7 till day 14).

This has been edited.
Throughout the figures, there are inappropriate staining for DAPI. The nuclear boundaries are not clear. Do you have any explanation for this?

This could be attributed to confocal acquisition parameters. We favored speed over quality to allow full thickness imaging. This generated speckled DAPI images but can be improved by adapting acquisition parameters.

Also, for MUC2, it is restricted to particular pole of the organoids, why it is not homogenous throughout the lumen?

We have observed this "incomplete" lumen expression of MUC2 in several organoid preparations.

In 2D FFPE sections from duodenum, goblet cells have a preferred location which also seems to be the case in our organoids and might at least partially explain this “incomplete” expression

We added a supplementary figure illustrating MUC2 staining in another organoid and in 2D duodenum biopsy section.

The same for LYZ, it appeared as you reduced the brightness and the images will be different when increasing the brightness.

LYZ staining appeared to be of lower intensity, although within the expected location. This explains the low signal-to-noise ratio.

**Reviewer #4**: The protocol presents an interesting and accessible option for researchers. While the manuscript offers valuable insights, including comparisons with the FLASH method, a more comprehensive evaluation is needed for a deeper understanding of its effectiveness. Comparing it with traditional techniques will further clarify its practical applications.
To strengthen the manuscript, I recommend adding comparisons between this protocol and results from traditional, slower methods, alongside FLASH.

This will help: provide essential context on the efficacy and reliability of your approach; highlight potential advantages and limitations, particularly regarding resolution and structural preservation. Visual comparisons across different methods will enhance understanding, and incorporating established protocols will better situate your findings within existing literature, bolstering the study’s credibility. Furthermore, it would be beneficial to explore whether this method is applicable to organoids other than those derived from the duodenal mucosa.

We now included a comparison of our workflow to more traditional immunostaining on 2d section of spheroid. “Compared to a traditional Immunostaining workflow on 2D sections of spheroids, we could avoid some challenging steps including: 1) the difficulty of finding organoids when sectioning, 2) time consuming 3) the lack of determining the level of the organoid/spheroid sectioned, 4) the limitation to acquiring only one layer (Charlet-Faure et al., 2023).”. **L269-273**

With regard to applicability to other organoids, tissue dissociation requires organ-specific dissociation solutions. However, the staining/imaging workflow described in this article should be applicable to any organoid type. Since we do not show here other organoids, we added this as a limitation of this study. **L291-L292**

**Reviewer #5**: Dear author the protocol presented add the microwave to demask antigens that could be a TIP to improve the antigen-antibody recognizing for this reason the protocol is not particular original

We agree with the reviewer that Heat induced epitope retrieval (HIER) is indeed routinely used for 2D FFPE sections. However, this cannot be extrapolated to 3D staining and, to the best of our knowledge, HIER is not well established for 3D staining due the complexity of the samples management. This sentence has been added in the introduction. **L58-L61**

could the authors add additional use of this protocol not only for imaging

The main aim of this protocol is imaging by confocal microscopy which is a crucial technique for analyzing enteroids. We did not test additional applications which should in theory be possible. We do not really want to speculate on this issue without solid proof and therefore prefer not to add any additional comment

however, some steps need to be clarified such as the step with citrate buffer at RT. Is the working solution at this step 10X?

This has been clarified (Fill each well with citrate buffer (1x) at RT). **L198**

Does Organoid Washing Buffer contain 1ml of Triton 100% with 2g BSA? please indicate in the step

This has been rephrased: 1L of PBS containing 1ml Triton X-100 and 2g BSA. **L210**

Lysozymes and MUC2 are not present images these markers are important because are intracellular to show if the antibody is able to go inside the cell

In case the complete figures have not been adequately up-loaded during our submission, we now up-loaded separately Figures 3A-B, C-D and E-F.

**Reviewer #6**: Re: 3D Fluorescence Staining and Imaging of Low Amount of Organoids: Protocol Accessible to All

Organoids are miniature tissues that exhibit the structure and specific functions of various organs. In recent years, with the advancement of organoid production techniques, these models have significantly contributed to the study and understanding of the mechanisms involved in the pathogenesis of various diseases. The ability to visualize organoids using three-dimensional fluorescence techniques in many cases, and the preparation of these organoids for imaging, are of crucial importance. The researchers who developed this protocol noted that in a microwave-based antigen retrieval method, which they term HIER, the target antigenic epitopes are unmasked, facilitating easier and more efficient labeling of the cells that compose the organoids. However, it is important to note that the microwave antigen retrieval method is not a novel technique, and it has not yielded consistent results across all antibodies they aimed to demonstrate. Additionally, I have further critiques and suggestions as outlined below.

We agree with the reviewer that Heat induced epitope retrieval (HIER) is indeed routinely used for 2D FFPE sections. However, this cannot be extrapolated to 3D staining and, to the best of our knowledge, HIER is not well established for 3D staining due the complexity of the samples management. Our protocol fills this gap showing its added value also for 3D staining. This sentence has been added in the introduction. **L58-L61**

Main Comments:

The background/introduction section of the protocol paper lacks sufficient citations. Certain statements sharing information and research results are not attributed to any specific study, despite not being original findings of this work.
In your results, you stated that no data were shown for MUC2, LYZ, and OLFM4 due to non-specific staining in intestinal organoids. However, OLFM4 staining is visible in Fig. 3. Furthermore, the figure legend of Fig. 3 indicates that OLFM4 is labeled in red in panels A and B, MUC2 is labeled in green in panels C and D, and LYZ is labeled in red in panels E and F. However, the figure file only contains panels A and B, where OLFM4 staining is visible. This discrepancy needs to be addressed and clarified.

This has been adapted. We agree that his is confusing and we apologize for the confusion.

Why did you perform imaging on a confocal microscope after tissue clearing? The resolution still appears to be low. Using light-sheet microscopy instead could improve image quality and better highlight the key steps in your protocol.

We totally agree that light-sheet imaging is a technique of choice for 3D imaging. It is much faster than confocal and has a very good axial resolution. However, when using samples as small as organoids, many of them are lost during the mounting step necessary to position them in the imaging chamber. For this reason, we do not find it suitable for small amount of tiny samples. Moreover, confocal imaging in multiwell plates allows an easy shift from growth follow-up with plate readers.

It is recommended to share the key parameters (e.g., exposure time, gain, etc.) used in the confocal imaging process.
The objective, detector and the lasers were added to Microscopes section. **L328-330**

Additionally, when referring to intestinal organoids, it is suggested to indicate "enteroids" in parentheses upon the first mention and to consistently use "enteroids" in subsequent sections.

This has been adapted.

**Reviewer #7**: The protocol is generally clear and well-written. The only major request I have is to indicate how many organoids have been successfully stained with this protocol.
We successfully stained as low as 10 organoids. This information has been integrated in the conclusion section. **L285**

Has this protocol been used in a published work?

Not yet, but it will be integrated into our future publications. In addition, PlosOne protocols require that protocols submitted for publication cannot be part of a finalized work and should be submitted upfront to study termination.

Please note that on line 146 timing is missing.

This has been filled in. **L113**

Please carefully check English spelling, e.g. line 17: “lost” should be replaced with “loss”, line 57:”brake” should be replaced with “break”

This has been corrected. **L56, L64**
